# Prognostic Biomarker KIF18A and Its Correlations With Immune Infiltrates and Mitosis in Glioma

**DOI:** 10.3389/fgene.2022.852049

**Published:** 2022-05-03

**Authors:** Bing-Yan Tao, Yu-Yang Liu, Hong-Yu Liu, Ze-Han Zhang, Yun-Qian Guan, Hui Wang, Ying Shi, Jun Zhang

**Affiliations:** ^1^ Medical School of Chinese PLA, Beijing, China; ^2^ Department of Neurosurgery, The First Medical Centre, Chinese PLA General Hospital, Beijing, China; ^3^ Department of Neurosurgery, Hainan Hospital of Chinese PLA General Hospital, Sanya, China; ^4^ Cell Therapy Center, Xuanwu Hospital, Capital Medical University, Beijing, China; ^5^ Department of Experimental Pathology, Beijing Institute of Radiation Medicine, Beijing, China; ^6^ School of Medicine, University of Electronic Science and Technology of China, Chengdu, China

**Keywords:** prognostic biomarker, Kif18A, immune infiltrates, glioma, bioinformatics

## Abstract

**Background:** Glioma is globally recognised as one of the most frequently occurring primary malignant brain tumours, making the identification of glioma biomarkers critically significant. The protein KIF18A (Kinesin Family Member 18A) is a member of the kinesin superfamily of microtubule-associated molecular motors and has been shown to participate in cell cycle and mitotic metaphase and anaphase. This is the first investigation into the expression of KIF18A and its prognostic value, potential biological functions, and effects on the immune system and mitosis in glioma patients.

**Methods:** Gene expression and clinicopathological analysis, enrichment analysis, and immune infiltration analysis were based on data obtained from The Cancer Genome Atlas (TCGA), with additional bioinformatics analyses performed. Statistical analysis was conducted in R software. Clinical samples were used to evaluate the expression of KIF18A via immunohistochemical staining. In addition, the expression level of KIF18A was validated on U87 cell line.

**Results:** Our results highlighted that KIF18A plays a key role as an independent prognostic factor in patients with glioma. KIF18A was highly expressed in glioma tissues, and KIF18A expression was associated with age, World Health Organization grade, isocitrate dehydrogenase (IDH) status, 1p/19q codeletion, primary therapy outcome, and overall survival (OS). Enrichment analysis revealed that KIF18A is closely correlated with the cell cycle and mitosis. Single sample gene set enrichment analysis (ssGSEA) analysis revealed that KIF18A expression was related to the immune microenvironment. The increased expression of KIF18A in glioma was verified in clinical samples and U87 cell line.

**Conclusion:** The identification of KIF18A as a new biomarker for glioma could help elucidate how changes in the glioma cell and immune microenvironment promote glioma malignancy. With further analysis, KIF18A may serve as an independent prognostic indicator for human glioma.

## Introduction

Glioma is the most common and aggressive malignancy in the brain, with high morbidity and mortality (Jiao et al., 2018). Originating from glial cells, glioma accounts for more than 30% of all brain tumours and over 80% of intracranial malignancy (Noorani et al., 2020). A recent review indicated the 5-years OS is only 4%, and the median overall survival is less than 2 years (Qu et al., 2020). Regarding glioblastoma, the most invasive type of glioma, median overall survival of patients decreased sharply to about 14 months (Weingart et al., 2007). Although multimodal treatment methods, such as chemotherapy, radiotherapy, and surgical resection, have been performed, the prognostic outcomes remain dismal (Wenger et al., 2020). Recently, many researchers have found that the mitotic activity is one of the important criterial for glioma grading and poor prognosis of glioma patients is related to uncontrolled mitosis of tumour cells (Perry et al., 2016; Rathore et al., 2020). The higher the mitotic activity of glioma cells, the higher the WHO grade of glioma, which tends to indicate a worse patient prognosis. Therefore, some biological factors associated with mitosis may act as novel therapeutic targets to improve patient outcomes.

As a member of the kinesin superfamily, KIF18A is a microtubule-associated molecular motor that utilises the hydrolysis of ATP to produce force and move along microtubules (Hirokawa et al., 2015). Previous studies have suggested that KIF18A is a microtubule depolymerase that inhibits the integral dynamics of the positive end of microtubules without disrupting their stability, and plays a significant role in aggregating chromosomes and maintaining chromosome stability during mitosis (Du et al., 2010). Recently, the expression and role of KIF18A in tumours have attracted increasing attention and have become a hotspot in molecular oncology research. It is noteworthy that some recent studies have revealed that KIF18A is closely related to the occurrence, development, invasion, and metastasis of various malignant tumours (Nagahara et al., 2011; Luo et al., 2018; Alfarsi et al., 2019). In addition, many researchers have reported that KIF18A is a potential therapeutic target and prognostic factor for a variety of tumours, including hepatocellular carcinoma, prostate cancer, and lung adenocarcinoma (Luo et al., 2018; Zhang et al., 2019; Zhong et al., 2019). However, the correlation and clinicopathological significance of KIF18A expression in glioma have not been studied, and further research is needed.

In this study, we performed an in-depth and all-inclusive bioinformatics analysis of KIF18A expression in gliomas according to some clinical datasets, and its role as a potential therapeutic target and independent prognostic factor was evaluated. We found that compared with normal tissues, KIF18A was highly expressed in glioma, and elevated expression of KIF18A was markedly associated with poor prognosis in patients with glioma. Moreover, analysis of genes and proteins that interact with KIF18A showed that several molecules related to cell cycle and cell division, such as RRP7A, ANAPC4, WEE1, CDC20, and NDC80, were enriched in glioma. In addition, functional enrichment analysis was conducted and the results implied that KIF18A differentially expressed genes (DEGs) were mainly enriched in cell cycle, DNA replication, microtubule cytoskeleton organization, and meiotic cell cycle, which further confirmed that KIF18A is involved in cell division. Finally, immune filtration analysis suggested that KIF18A might be able to regulate the immune microenvironment of gliomas. In summary, KIF18A may be a potential biomarker and a promising therapeutic target for gliomas.

## Materials and Methods

### Gene Expression Analysis

RNA-seq data and corresponding clinical information of glioma in level 3 HTSEQ-FPKM format were downloaded from the TCGA database (Tomczak et al., 2015), converted to TPM (Transcripts Per Million reads) format, and converted into log2 format for further analysis. Statistical analysis was performed using R software v3.6.3, and statistical significance was set at *p* < 0.05.

### Culture of Human Glioblastoma and Astrocyte Cell Lines

U87 (human glioblastoma cell line) and SVG (human astrocyte cell line) were purchased from the Institute of Basic Medicine, China Medical College. Two kinds of cell lines were cultured in Dulbecco’s modified Eagle’s medium (DMEM) containing with 10% fetal bovine serum at 37°C under an atmosphere of 5% CO_2_.

### Glioma Sample Collection

Glioma samples were collected from Department of Neurosurgery, PLA General Hospital. Seventeen paraffin-embedded samples (1 case was normal brain tissue, 2 cases were grade 2, 4 cases were grade 3 and 10 cases were grade 4) were used for immunohistochemistry staining. The patient was informed of the content of this research and signed an informed consent before the operation. The study was approved by the Institutional Research Ethics Committee of the PLA General Hospital (batch number: S2018-089-01).

### KIF18A Expression in Different Tumour Types and Glioma

We investigated KIF18A expression in different tumour types from the TCGA database, which combined various types of information to analyze the role of KIF18A in multifarious tumours. We also investigated the expression of KIF18A in glioma and its subtypes (low-grade glioma [LGG] and glioblastoma [GBM]). In addition, The Human Protein Atlas (HPA) database (Pontén et al., 2008) was utilised to analyze the available immunohistochemical images. The R software v3.6.3 was utilised to perform the statistical analysis, and visualisation was implemented using the ggplots2 package v3.3.3.

### Correlations Between the KIF18A Expression and Diverse Clinical Characteristics

Box plots are generated to examine the correlations of KIF18A expression with diverse clinical characteristics (age, WHO grade, IDH status, 1p/19q codeletion, primary therapy outcome, and OS event) in patients. The R software v3.6.3 was utilised to perform the statistical analysis, and visualisation was implemented using the ggplots2 package v3.3.3.

### Functional Enrichment Analysis of DEGs

Volcano plots and heat maps were used to visualise the results of the top DEGs. The R software v3.6.3 was utilised to perform the statistical analysis, and visualisation was implemented using the ggplots2 package v3.3.3. Additionally, the interaction network of KIF18A-relative biological function was analyzed using the Metascape database (Zhou et al., 2019).

### Tumour Immune Microenvironment Analysis

To deepen our understanding of the landscape of the KIF18A-related immune microenvironment, the immune and stromal scores, ESTIMATE scores, and immune cell infiltration were calculated and compared between the KIF18A-high and KIF18A-low groups. Statistical analysis was performed using Estimate package v1.0.13. In addition, the enrichment score of 24 immune cells was evaluated based on ssGSEA algorithms (Hänzelmann et al., 2013) utilizing the GSVA package v1.34.0. This package was also used to perform correlation analysis between immune infiltration and KIF18A expression levels.

### Survival Prognosis Analysis

Kaplan-Meier plots were used to evaluate the relationship between KIF18A expression and OS of patients. In addition, a time-dependent receiver operating characteristic (ROC) curve was constructed for KIF18A expression in gliomas. The R software v3.6.3 was used to perform the statistical analysis, and visualisation was accomplished using the Survminer package v0.4.9 version. The multivariate Cox regression was performed in the hypothesis test, we used the R software v3.6.3 to perform the statistical analysis, and accomplished visualisation using the survival package v3.2-10.

### Analysis of KIF18A-Interacting Molecules and Functional Enrichment

The KIF18A associated gene-gene interaction network was established using the GeneMANIA database (WardeFarley et al., 2010). The KIF18A-related protein-protein interaction (PPI) network was established using the STRING online database (Szklarczyk et al., 2021). Gene Ontology (GO) and Kyoto Encyclopedia of Genes and Genomes (KEGG) enrichment analyzes were performed for the top 20 KIF18A-binding proteins. The R cluster Profiler package v3.14.3 was used for statistical analysis, and the ggplot2 package v3.3.3 was used for visualisation.

### Immunohistochemistry

Tissues were fixed in 4% paraformaldehyde, embedded in paraffin, and cut into 4 μm slices. These slices were placed on slides and processed as previously described. The tissue slices were incubated with primary anti-KIF18A (1:1000, 19245-1-AP, Proteintech) antibody diluted with 1% goat serum (Balb, WE0320) in PBS overnight. Tissue slices were incubated with the secondary antibody for 1 h. Then, the ABC Horseradish Peroxidase kit (Vector Laboratories) was used to stain the slices and visualisation was accomplished using DAB (3,3′-diaminobenzidine). Nuclei were counterstained with haematoxylin. The immunohistochemical staining results were analyzed and scored by two pathologists who were blinded to the sources of the clinical samples. The intensity of staining was analyzed by the semiquantitative integration method.

### Western Blot

Equal amounts of protein lysates (30 μg) of two kinds of cell line were separated on 10% SDS-PAGE gels and transferred into a PVDF membrane (0.45µm; Amersham Bioscience, Freiburg, Germany). After blocking with milk, the membrane was incubated with primary antibodies KIF18A (19245-1-AP, Proteintech, Wuhan, China) and GAPDH (10494-1-AP, Proteintech, Wuhan, China).

### Statistical Analysis

For bioinformatics analysis, the statistical significance between two groups was detected by using the Wilcoxon rank sum test, and the Kruskal-Wallis test and Dunn’s tests were utilised for comparison of multi-groups. The correlation between KIF18A expression and other immune-relevant genes was calculated and evaluated by Spearman’s correlation coefficient. All statistical analysis was performed using R software (version 3.6.3), and two-tail *p* < 0.05 was considered as of statistical significance.

## Results

### KIF18A Is Highly Expressed in Glioma

**FIGURE 1:**
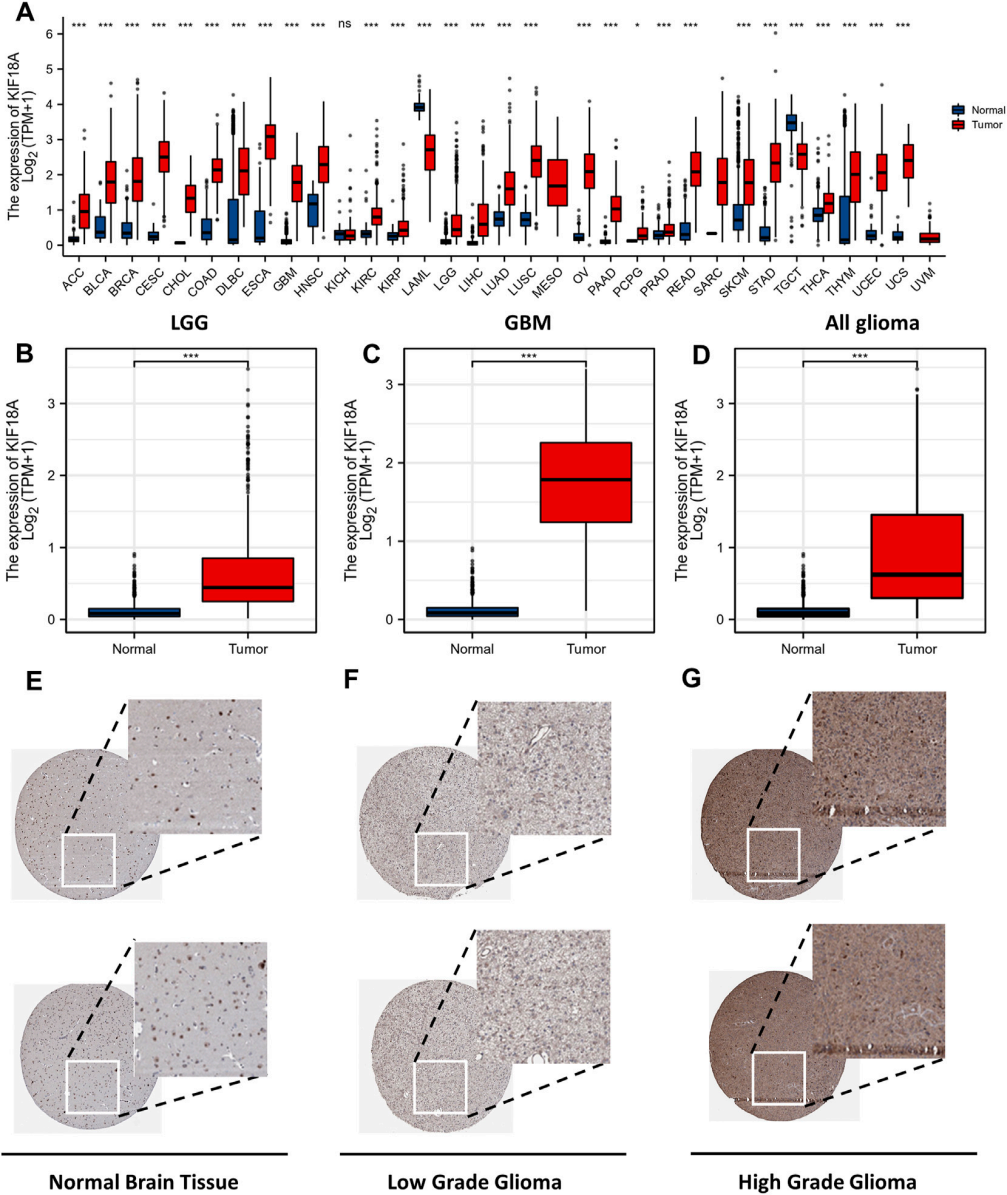
KIF18A mRNA expression level in tumours and normal tissue. The mRNA expression level of KIF18A in **(A)** different tumour types, **(B)** LGG, **(C)** GBM, **(D)** All glioma was investigated with TCGA database. Cerebral expression of KIF18A protein in Normal Brain Tissue **(E)**, Low Grade Glioma **(F)**, High Grade Glioma **(G)** was visualized using immunohistochemistry via the HPA. (ns, *p* ≥ 0.05; *p < 0.05, **p < 0.01, ***p < 0.001).

The analysis of KIF18A expression in different tumour types from the TCGA database revealed that apart from acute myeloid leukemia (AML), kidney chromophobe (KICH), and testicular germ cell tumours (TGCT), KIF18A was highly expressed in nearly all the tumours when compared with normal tissue (Figure 1A). In addition, the expression of KIF18A in GBM, LGGs and all gliomas was significantly higher than that in normal brain tissue (Figures 1B–D **)**. We then analyzed KIF18A protein expression using the HPA database and showed the immunohistochemical staining results in Figures 1E–G. Taken together, glioma displays prominently higher KIF18A expression than that in normal brain tissue at both the mRNA and protein levels.

### KIF18A Expression Is Correlated With Different Clinical Characteristics

**FIGURE 2:**
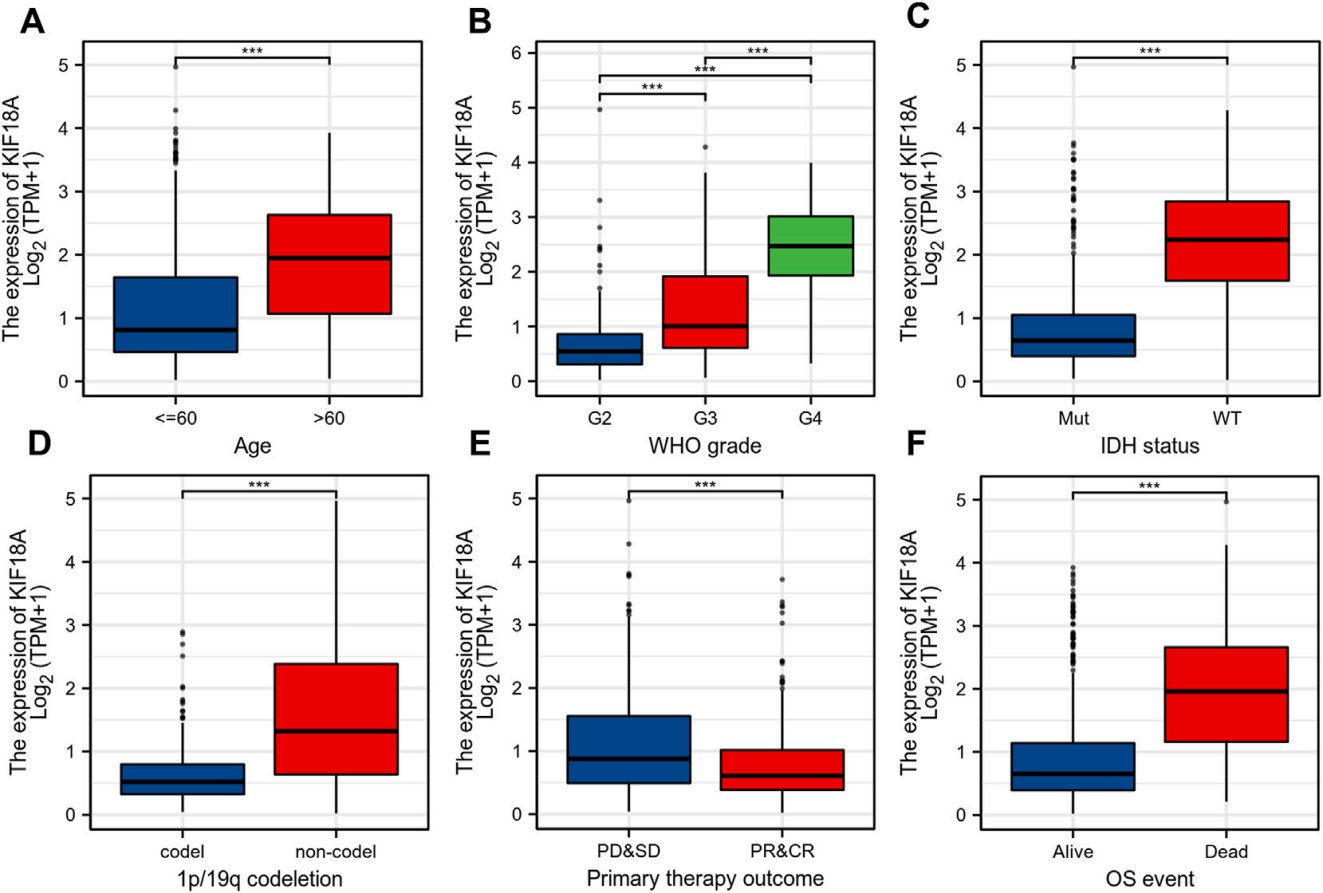
Correlations between the KIF18A mRNA expression and different indicators of poor clinical prognosis of glioma patients. **(A)** Age, **(B)** World Health Organization (WHO) grade, **(C)** IDH status, **(D)** 1p/19q codeletion, **(E)** primary therapy outcome, **(F)** OS event. (***p < 0.001).

To further clarify the role of KIF18A in glioma, we analyzed the mRNA expression of KIF18A in different clinical subgroups. First, KIF18A mRNA expression levels were increased in patients over 60 years of age (Figure 2A). In addition, the expression level of KIF18A increased with an increase in tumour grade, and the highest expression level of KIF18A was found in grade 4, the highest degree of malignancy (Figure 2B). According to the IDH status, the expression of KIF18A was significantly higher in wild-type patients than in mutant patients (Figure 2C). Lower expression of KIF18A was found in the 1p/19q codeletion group compared to that in the non-codeletion group (Figure 2D). In addition, KIF18A expression was significantly lower in patients with partial response and complete response than in patients with other primary treatment outcomes (Figure 2E). In terms of OS, KIF18A mRNA expression levels in the dead group were higher than those in the surviving group (Figure 2F). In summary, KIF18A mRNA expression is related to the clinical characteristics of glioma, and its high expression might suggest a poor prognosis.

### DEGs Between High and Low KIF18A-Expressing Groups in Glioma

**FIGURE 3:**
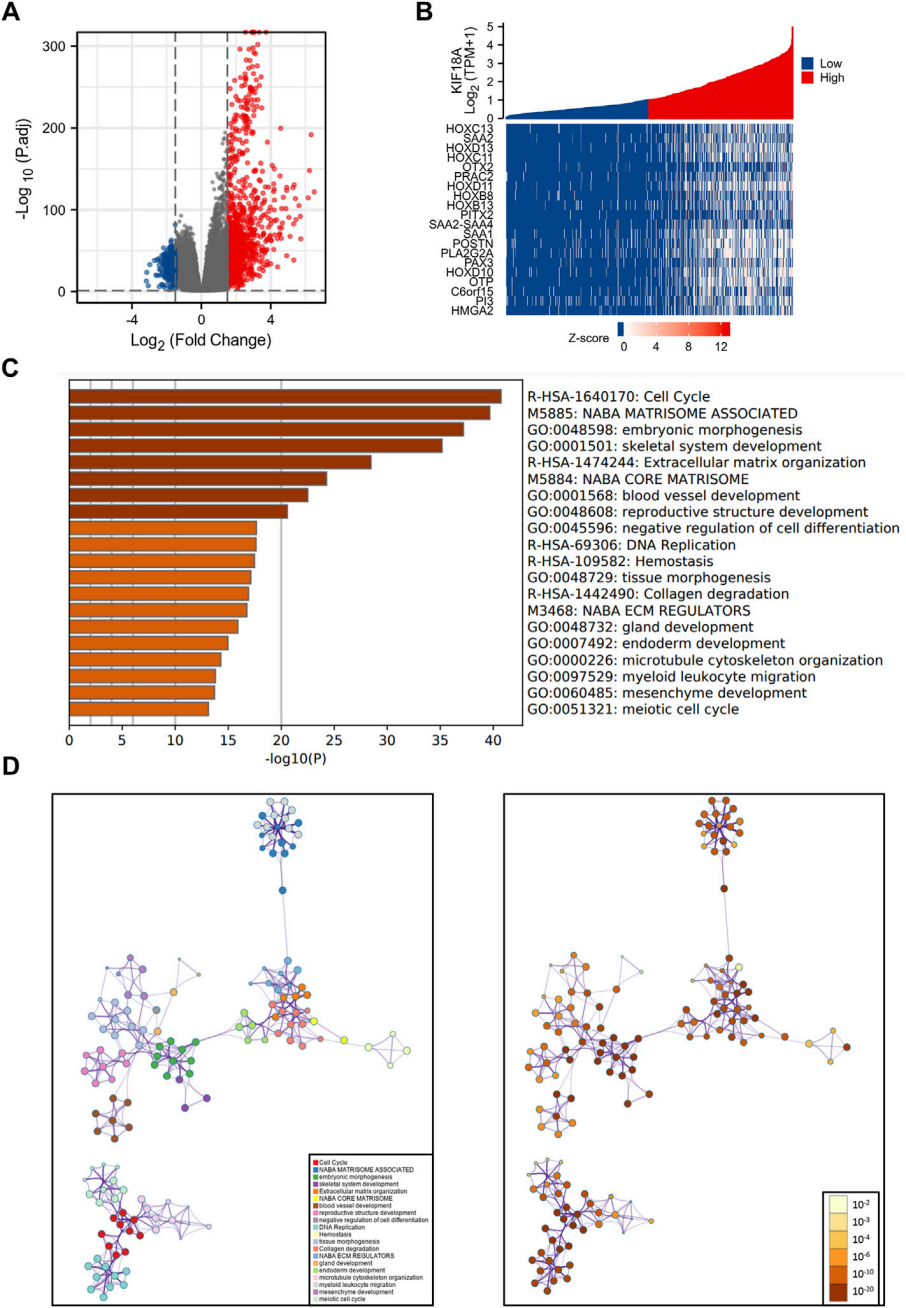
Visualization and functional enrichment analysis of the DEGs between high and low KIF18A expression groups. **(A)** Volcano plot of the top DEGs. **(B)** Heat maps of the top 20 DEGs. **(C)** Bar plot of 20 most relative biological functions enriched terms. **(D)** Interaction network of 20 most relative biological functions.

According to the threshold values of |log2 fold-change (FC)|>1.5 and adjusted *p*-value<0.05, 1233 DEGs were obtained, including 952 upregulated genes and 281 downregulated genes (Figure 3A). The heat maps showed the top 20 DEGs, including HOXC13, SAA2, HOXD13, HOXC11, and OTX2 (Figure 3B). In addition, functional enrichment analysis was conducted to identify the most relevant biological functions of these DEGs, which revealed that DEGs were mainly enriched in functions related to cell division, including cell cycle, DNA replication, microtubule cytoskeleton organization, and meiotic cell cycle (Figure 3C). To further capture the correlation between these enriched biological functions, we mapped them into a network plot using metascape online database, as shown in Figure 3D. And the specific information of important nodes related to cell division in the network was shown in detail in the Supplementary Table S1.

### KIF18A-Correlated Immune Cells Infiltration Analysis

**FIGURE 4:**
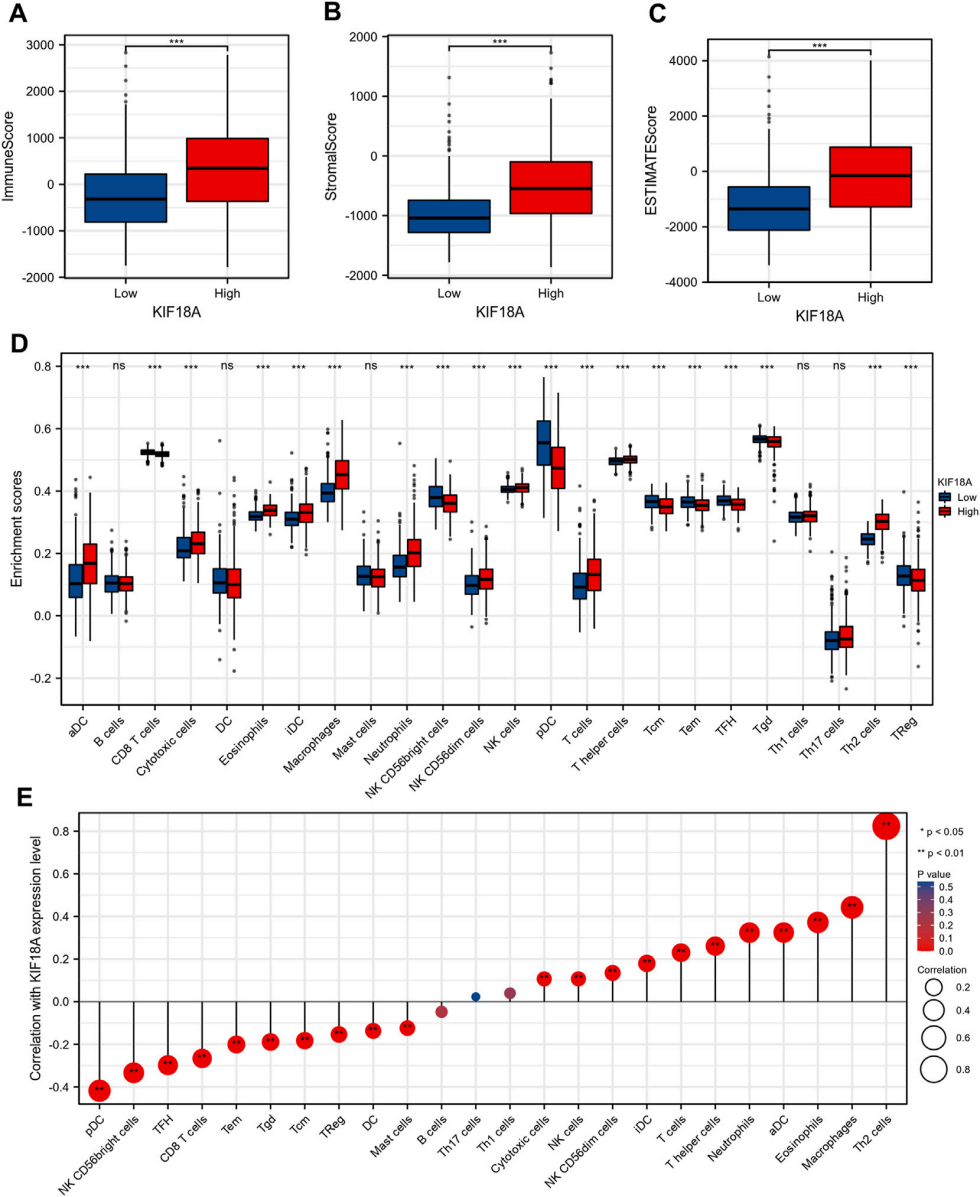
The correlations between KIF18A mRNA expression level and immune cell infiltration. Comparison of Immune Score **(A)**, Stromal Score **(B)** and ESTIMATE Score **(C)** between the KIF18A-high and KIF18A-low patient groups. **(D)** The varied proportions of 24 subtypes of immune cells in high and low KIF18A expression groups. **(E)** The correlations between KIF18A mRNA expression level and the infiltration of different subtypes of immune cells. (ns, *p* ≥ 0.05; *p < 0.05, **p < 0.01, ***p < 0.001).

To further investigate the mechanism underlying differences in survival between the KIF18A-high and KIF18A-low expression groups, we analyzed the correlation between KIF18A expression level and tumour microenvironment. According to the TCGA database, patients with elevated KIF18A expression tended to have markedly higher immune score (Figure 4A), stromal score (Figure 4B), and estimate score (Figure 4C). Enrichment analysis of immune infiltrating cells was performed, and the results revealed that aDC, cytotoxic cells, eosinophilis, iDC, macrophages, neutrophils, CD56dim cells, NK cells, T cells, T helper cells, and Th2 cells were clearly increased in the microenvironment of the KIF18A-high group (Figure 4D). In addition, we studied the correlation between multifarious immune infiltration cells and KIF18A expression levels, and found that Th2 cells, macrophages, eosinophils, aDC, and neutrophils were significantly positively correlated with KIF18A expression. In contrast, pDC, NK CD56bright cells, TFH, CD8 T cells, and Tem were markedly negatively correlated with the expression of KIF18A (Figure 4E). In summary, the expression of KIF18A may reflect immune infiltration status.

### Predictive Performance and Independent Prognostic Value of KIF18A Expression

**FIGURE 5:**
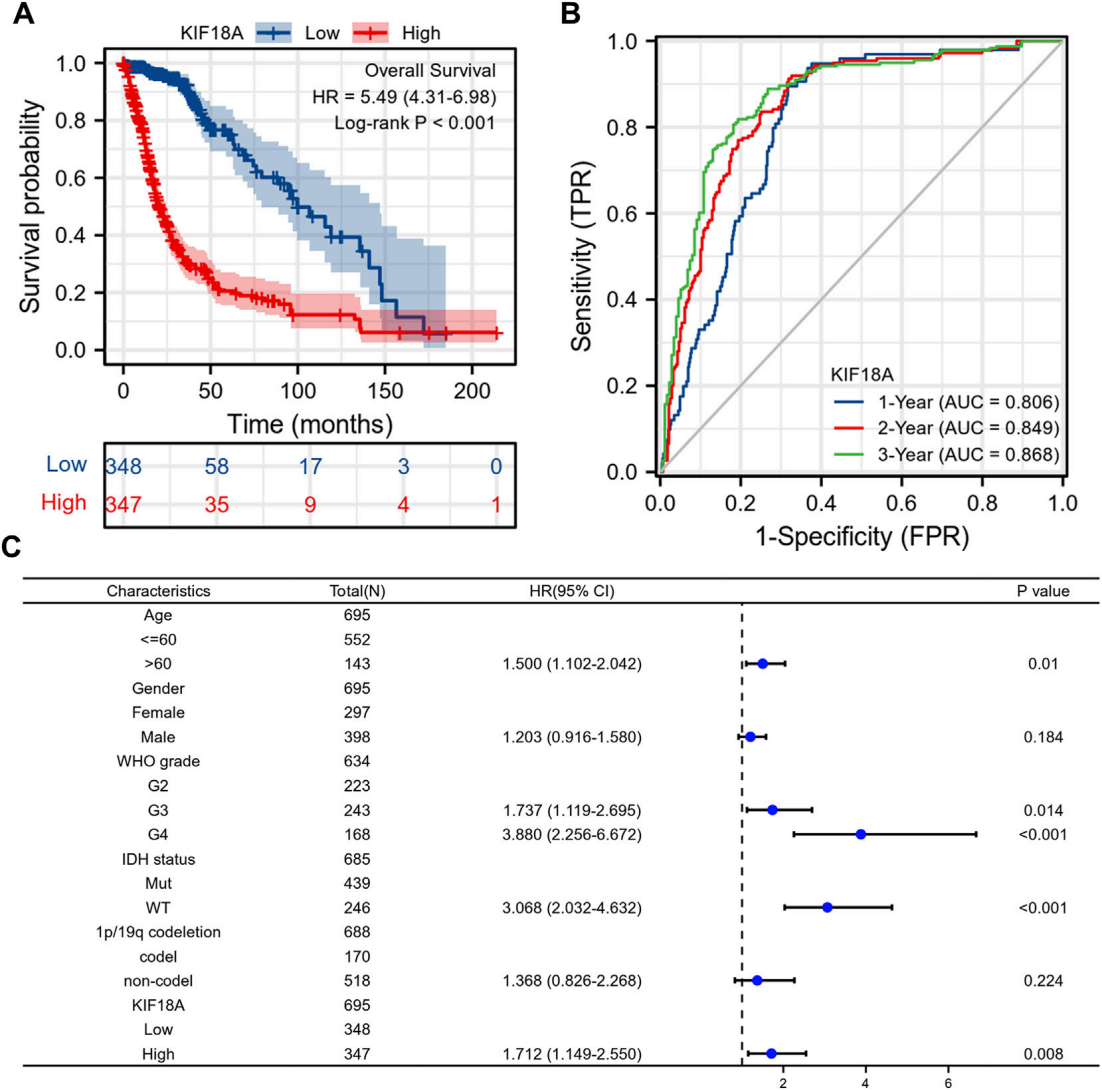
The correlations between KIF18A mRNA expression level and the overall survival rate of patients. **(A)** Kaplan–Meier plots of KIF18A; **(B)** time-dependent ROC curves of KIF18A. **(C)** Multivariate Cox analysis of KIF18A expression and clinicopathological characteristics.

As previously mentioned, KIF18A expression was significantly associated with the malignant characteristics of gliomas. We then investigated the prognostic value of the KIF18A expression level. According to Kaplan-Meier plots, patients with higher KIF18A expression had worse OS (Figure 5A). The time-dependent ROC curve showed that the expression level of KIF18A had a good predictive effect on glioma survival (Figure 5B). In Multivariate Cox regression analysis, the elevated expression of KIF18A was found to be an independent risk factor for poor prognosis in glioma patients (Figure 5C). In other words, the higher the expression of KIF18A, the worse the prognosis.

### KIF18A-Interacting Genes and Proteins

**FIGURE 6:**
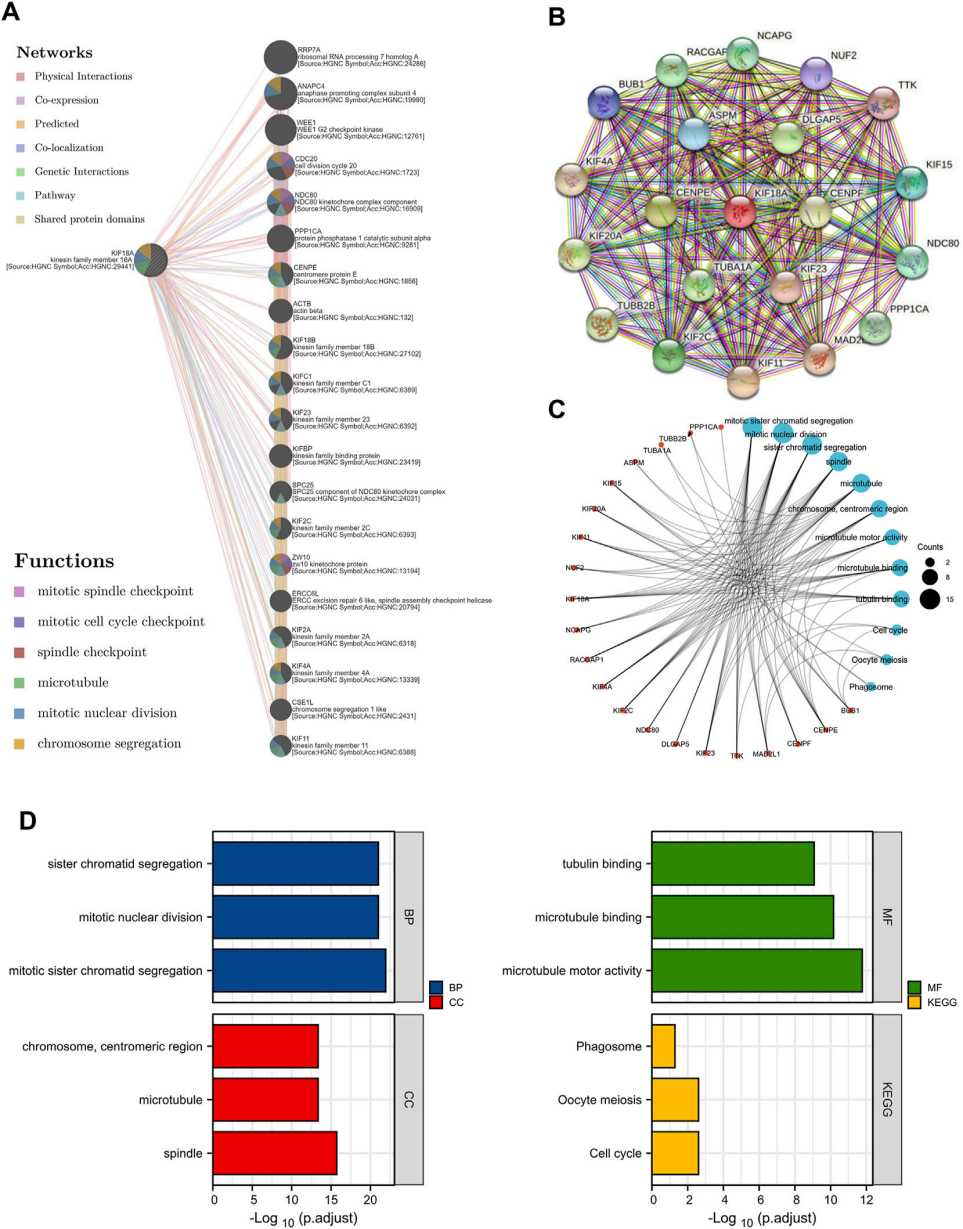
Gene-gene interaction network, PPI network and enrichment analysis related to binding proteins of KIF18A. **(A)** KIF18A associated gene-gene interaction network. **(B)** KIF18A-related PPI network. **(C)** Enrichment analysis network of KIF18A-related molecules. **(D)** GO and KEGG analysis.

To further assess the function of KIF18A, we investigated the genes and proteins interacting with KIF18A. First, a gene-gene interaction network was automatically constructed to identify the top 20 relevant genes associated with KIF18A utilising GeneMANIA online database, including RRP7A, ANAPC4, WEE1, CDC20, and NDC80, and most of them were related to the cell cycle and cell division (Figure 6A). In addition, according to STRING online database, a protein-protein interaction network was constructed, and the top 20 proteins relevant to KIF18A were identified (Figure 6B). We further analyzed these KIF18A binding proteins and their corresponding biological functions (Figure 6C). According to GO and KEGG enrichment analysis (Figure 6D), the results revealed that the primary biological processes (BP) included sister chromatid segregation, mitotic nuclear division, and mitotic sister chromatid segregation. The cellular component (CC) was primarily enriched in the chromosome, centromeric region, microtubule, and spindle. The molecular function (MF) was mainly involved in tubulin binding, microtubule binding, and microtubule motor activity. KEGG pathway enrichment was mainly related to phagosomes, oocyte meiosis, and cell cycle.

### Validation of KIF18A Expression in Clinical Samples and Cell Lines

**FIGURE 7:**
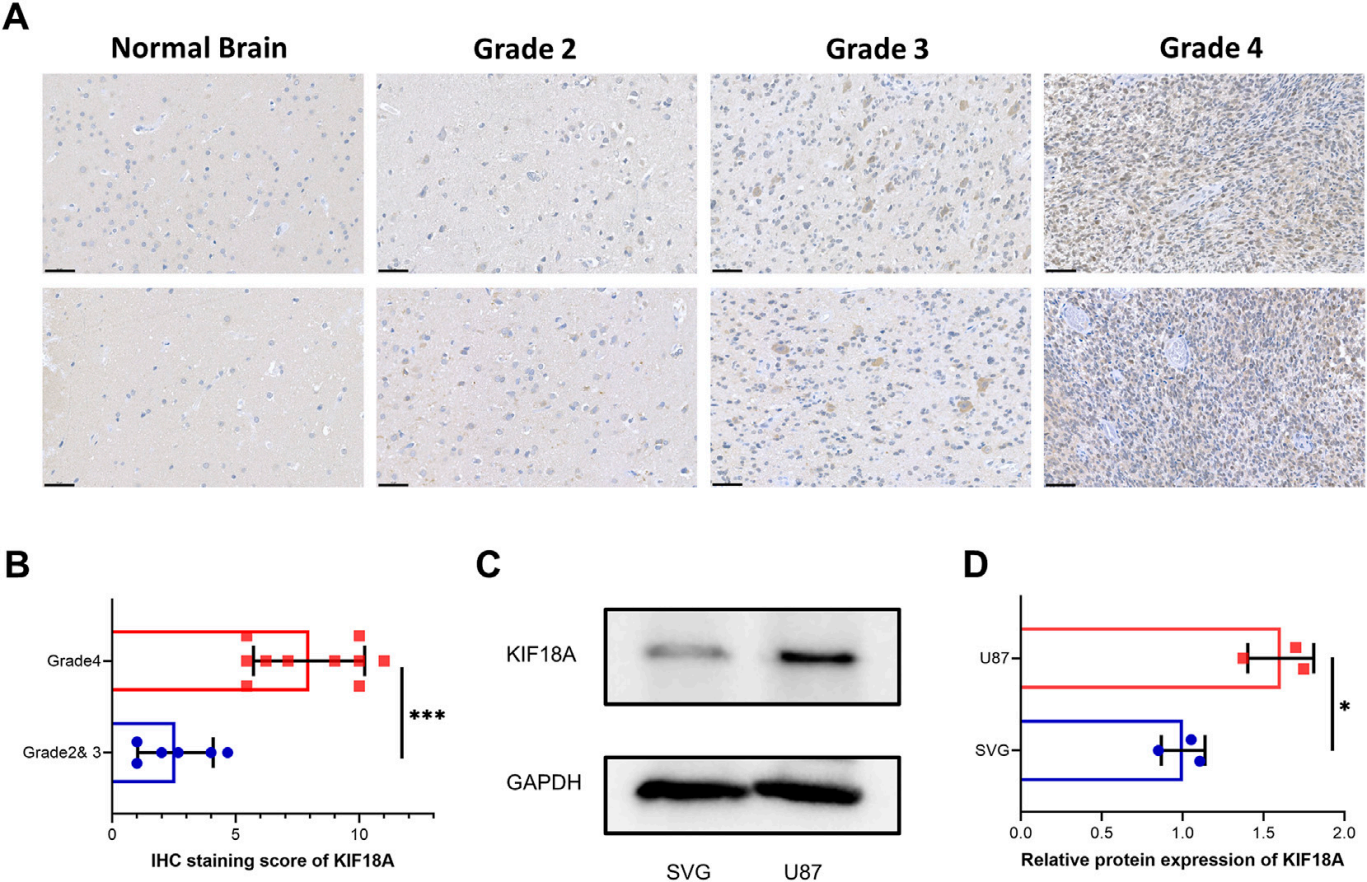
Validation of KIF18A expression in clinical samples and cell lines. **(A)** Confirmation of KIF18A expression using immunohistochemical staining in normal brain and glioma tissues. Scare bars, 50 μM. **(B)** IHC staining score of KIF18A in glioma with different grade. **(C)** Examination of the expression of KIF18A in the U87 and SVG cell line by western blot. **(D)** Quantitative data of KIF18A in the U87 and SVG cell line. (*p < 0.05, **p < 0.01, ***p < 0.001).

To verify the above results obtained from multifarious databases, we collected seventeen paraffin-embedded samples from patients with glioma, and immunohistochemical tests were performed on these samples. The results suggested that patients with higher grade of glioma tend to have more KIF18A expression (Figures 7A, B). In addition, according to western blot, we found that compared with the SVG cell line, KIF18A was highly expressed in the U87 cell line (Figures 7C, D). In conclusion, the experiment results verified by clinical samples and cell lines was consistent with the data from bioinformatics.

## Discussion

Glioma is the most common type of malignant brain tumour. Glioma cells are extremely invasive, and patients with glioma have a short survival time and poor prognosis (Xiong et al., 2015). However, at present, the overall treatment effect is unsatisfactory; thus, identifying new biomarkers is of great significance (Nie et al., 2020). Previous studies have found that malignant proliferation of gliomas may be associated with uncontrolled mitosis (Christoforidis et al., 2012). Therefore, we investigated new molecular targets that regulate mitosis in order to improve patient prognosis.

As a member of the kinesin superfamily, KIF18A is a microtubule depolymerase that binds to microtubules, and utilises the energy generated by ATP hydrolysis to move along the cytoskeleton to ensure smooth intracellular transport and mitotic processes (Walczak et al., 2013). Recently, an increasing number of scholars have paid attention to the role of KIF18A in tumours, in order to investigate the mechanism of involvement of KIF18A in tumour genesis, development, and evolution, and to provide a basis for KIF18A as a molecular target for tumour therapy. Several studies have suggested that the level of KIF18A expression is strongly related to mitosis. Abnormal mitosis may lead to a variety of adverse consequences, such as gene mutations, apoptosis, and even tumorigenesis (Morfini et al., 2016). It was found that KIF18A depletion resulted in mitotic arrest and blocking KIF18A could affect the cell cycle and normal mitosis, leading to stagnation of mitosis, while the number of mitotic cells containing multipolar spindles increased significantly when KIF18A was overexpressed (Catarinella et al., 2009; Du et al., 2010; Braun et al., 2015; Janssen et al., 2018). In other words, either high or low expression of KIF18A can lead to abnormal chromosomal regulation processes, resulting in chromosome instability or affecting chromosome separation. In view of the key role of KIF18A in chromosome stabilisation and cell division, KIF18A is likely to be an important molecular target for abnormal cell proliferation and carcinogenesis. Moreover, in recent years, accumulating evidence has demonstrated that KIF18A is highly expressed in a variety of human malignant tumours and is widely involved in the occurrence, development, and outcome of tumours (Marra et al., 2013; EichenlaubRitter, 2015). In addition, some studies have found that KIF18A expression may be related to the prognosis of patients with glioma (Cho et al., 2019). However, in this study, we further investigated whether KIF18A is an independent risk factor affecting the prognosis of glioma, and explored the relationship between KIF18A expression and immune cells infiltration. Herein, we found that KIF18A was highly expressed in glioma, and then we constructed gene-gene interactions and protein-protein interaction networks to identify the molecules most related to KIF18A, including RRP7A, ANAPC4, WEE1, CDC20, and NDC80, and we found that most of them were associated with the cell cycle and cell division. In addition, enrichment analysis suggested that there were significant differences in cell cycle and DNA replication between high and low KIF18A expression groups. The above studies further validated the role of KIF18A in mitosis and tumorigenesis based on previous studies.

Additionally, studies have shown that the tumour-suppressive immune microenvironment is also an important factor for poor prognosis in glioma patients (Suzuki et al., 2020). To deeply explore the potential biological functions of KIF18A and further explain why high expression of KIF18A is associated with poor prognosis of patients, we conducted immune infiltration analysis based on the ssGSEA algorithm; the results indicated that the expression level of KIF18A was prominently correlated with Th2 cells and tumour-associated macrophages (TAMs), and the numbers of these two kinds of cells in the patient group with high expression of KIF18A were both highly expressed with significant statistical differences.

Th2 is one of the special subtypes of CD4^+^ helper T cell (Th cell) that mainly secretes cytokines such as IL-4, IL-5, and IL-10, and excessive Th2 cells could affect the tumour-suppressive immune microenvironment, seriously reducing the anti-tumour effect, leading to the malignant growth of tumours (Chen et al., 2013; Fang et al., 2018). Previous studies have revealed that many tumours, including lung cancer, glioma, breast cancer, and colorectal cancer, have Th1/Th2 balance drift in the immune system, and Th2 cells are often dominant, which might be related to the immune escape of tumours. Simultaneously, the increase in Th2 cells was positively correlated with the degree of malignancy (Shimato et al., 2012; Almatroodi et al., 2016; Faucheux et al., 2019; Scott et al., 2021). Taken together, we speculate that the abnormal increase in Th2 cells in the tumour microenvironment might be one of the crucial factors for the poor prognosis of patients with high expression of KIF18A.

TAMs are an important part of the glioma immune microenvironment, accounting for 30–50% of the total cells in solid tumours (Hambardzumyan et al., 2016). TAMs can be divided into two major subtypes (M1 and M2), and M1 plays an anti-tumour role, while M2 promotes tumour growth and invasion (Okubo et al., 2021). According to previous studies, most TAMs in the glioma immune microenvironment have the M2 phenotype, which indicates that they may play a significant role in glioma immune escape, recurrence, drug resistance, and malignant transformation (Huang et al., 2017). Meanwhile, our study has suggested that in the KIF18A high expression group, macrophages were significantly recruited into the glioma immune microenvironment. Therefore, we speculated that the expression level of KIF18A was correlated with the immune infiltration of TAMs, which might be another mechanism regulating the prognosis of patients.

In addition, there was an interaction between Th2 cells and macrophages in tumour immune microenvironment. Macrophages secrete TGF-β and IL-10 to convert Th1 cells into Th2 cells, thus reversing the anti-tumour effects of CD8^+^ cytotoxic T cells and CD4+Th1 cells. While Th2 cells secrete IL-4, IL-5, and IL-10 to promote polarisation of M2 macrophages, the positive interaction between the 2 cells constructs a tumour-suppressive immune microenvironment (Qiu et al., 2021). In conclusion, patients with high expression of KIF18A tend to have more inhibitory immune cell infiltration, and KIF18A may play an important role in the construction of a tumour-suppressive immune microenvironment.

This study had several limitations. First, most of the data in this study were obtained from open-source databases, and follow-up experiments are needed for further verification. Second, in this study, we mainly focused on the correlation between KIF18A and Th2 cells and TAMs, and further study of the relationship between KIF18A and other infiltrating immune cells is needed. In conclusion, our results suggest that KIF18A may be useful as a new prognostic indicator for gliomas. However, other underlying mechanisms remain largely unknown, and further studies are needed.

## Conclusion

In conclusion, our results suggested that KIF18A might affect the prognostic of glioma patients through its involvement in mitosis and regulation in immune cell infiltration. KIF18A might be a new biomarker and therapeutic target for glioma patients.

## Data Availability

The original contributions presented in the study are included in the article/Supplementary Material, further inquiries can be directed to the corresponding author.
