# Supplementary figures and images for: Prognostic Biomarker KIF18A and Its Correlations With Immune Infiltrates and Mitosis in Glioma
Source: Front Genet. 2022 May 3;13:852049. doi: 10.3389/fgene.2022.852049 (PMC9110815; doi:10.3389/fgene.2022.852049)

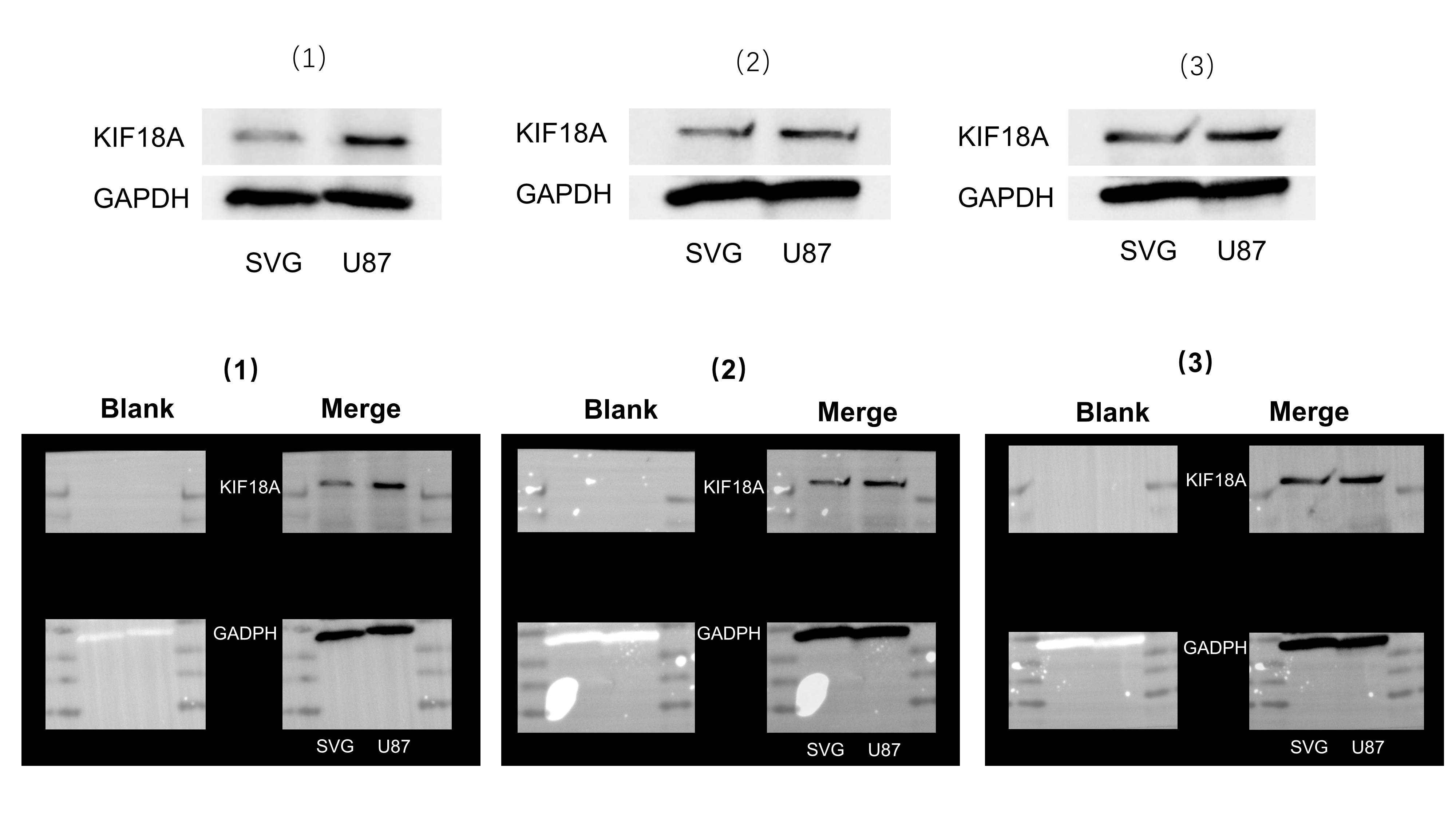

Supplement: Supplementary file 1 [file DataSheet1.ZIP › Raw dataú¿KIF18Aú⌐/WB/quantitive data of immunobloting.tif]

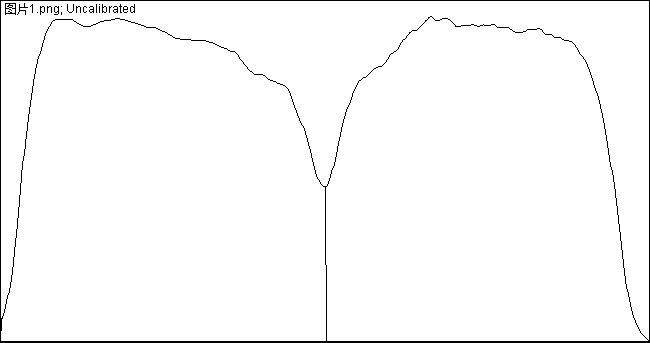

Supplement: Supplementary file 1 [file DataSheet1.ZIP › Raw dataú¿KIF18Aú⌐/WB/quantitive raw data/1/Plots of ═╝╞1⁄41-GAPDH.tif]

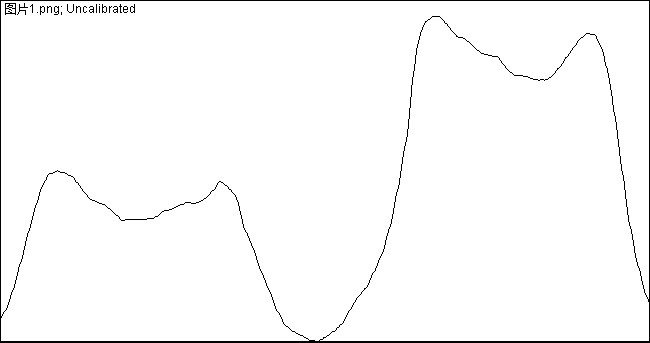

Supplement: Supplementary file 1 [file DataSheet1.ZIP › Raw dataú¿KIF18Aú⌐/WB/quantitive raw data/1/Plots of ═╝╞1⁄41-kif18A.tif]

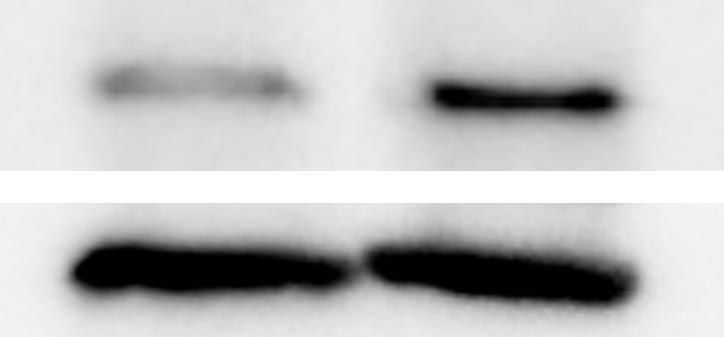

Supplement: Supplementary file 1 [file DataSheet1.ZIP › Raw dataú¿KIF18Aú⌐/WB/quantitive raw data/1/═╝╞1⁄41.png]

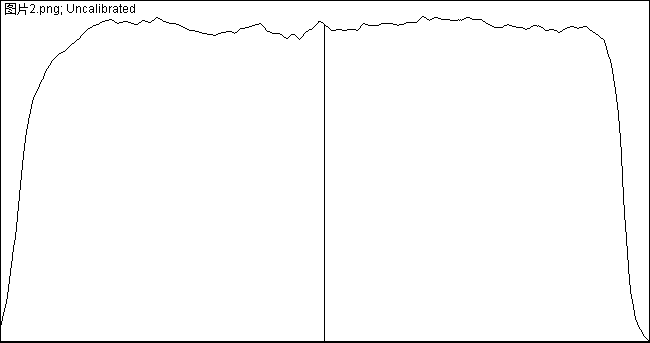

Supplement: Supplementary file 1 [file DataSheet1.ZIP › Raw dataú¿KIF18Aú⌐/WB/quantitive raw data/2/Plots of ═╝╞1⁄42-GADPH.tif]

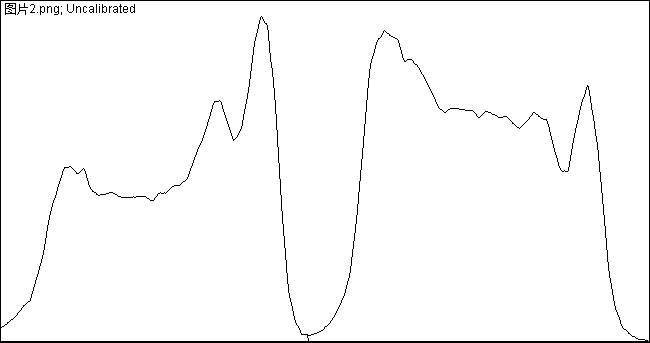

Supplement: Supplementary file 1 [file DataSheet1.ZIP › Raw dataú¿KIF18Aú⌐/WB/quantitive raw data/2/Plots of ═╝╞1⁄42-KIF18A.tif]

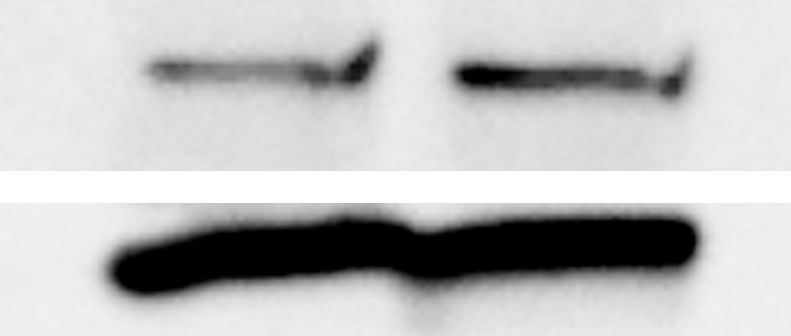

Supplement: Supplementary file 1 [file DataSheet1.ZIP › Raw dataú¿KIF18Aú⌐/WB/quantitive raw data/2/═╝╞1⁄42.png]

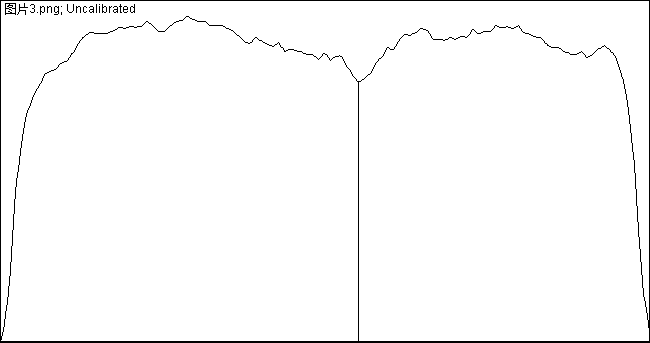

Supplement: Supplementary file 1 [file DataSheet1.ZIP › Raw dataú¿KIF18Aú⌐/WB/quantitive raw data/3/Plots of ═╝╞1⁄43.-GADPHtif.tif]

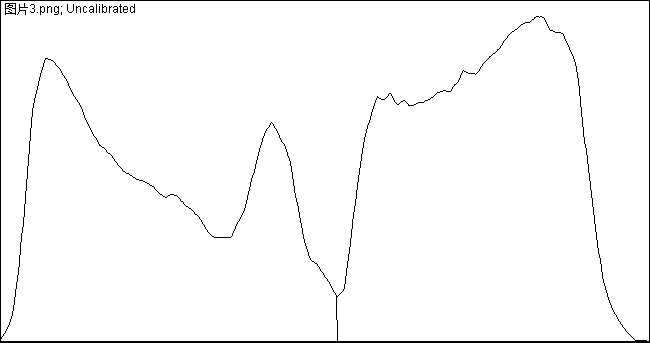

Supplement: Supplementary file 1 [file DataSheet1.ZIP › Raw dataú¿KIF18Aú⌐/WB/quantitive raw data/3/Plots of ═╝╞1⁄43-KIF18A.tif]

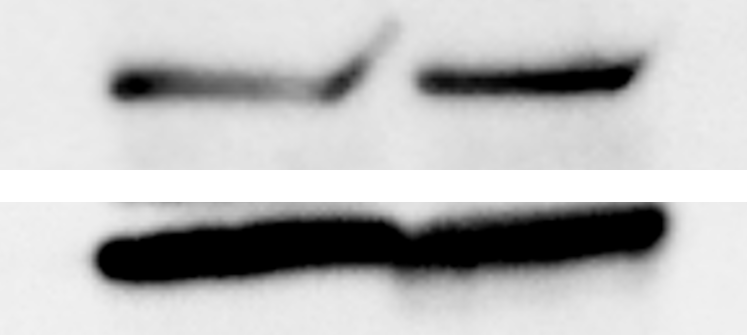

Supplement: Supplementary file 1 [file DataSheet1.ZIP › Raw dataú¿KIF18Aú⌐/WB/quantitive raw data/3/═╝╞1⁄43.png]
